# Importance of Certain Varieties of Cucurbits in Enhancing Health: A Review

**DOI:** 10.3390/foods13081142

**Published:** 2024-04-09

**Authors:** Jaqueline Romo-Tovar, Ruth Belmares Cerda, Mónica L. Chávez-González, Rosa M. Rodríguez-Jasso, Sonia A. Lozano-Sepulveda, Mayela Govea-Salas, Araceli Loredo-Treviño

**Affiliations:** 1 Food Research Department, School of Chemistry, Universidad Autónoma de Coahuila, Saltillo 25280, Mexico; j.romo@uadec.edu.mx (J.R.-T.); ruthbelmares@uadec.edu.mx (R.B.C.); monicachavez@uadec.edu.mx (M.L.C.-G.); rrodriguezjasso@uadec.edu.mx (R.M.R.-J.); m.govea.salas@uadec.edu.mx (M.G.-S.); 2 Department of Biochemistry and Molecular Medicine, School of Medicine, Universidad Autónoma de Nuevo León, Monterrey 64460, Mexico

**Keywords:** cucurbitaceae, therapeutic uses, bioactivities, nutritional health

## Abstract

The Cucurbitaceae family is an extensive group of fruits and vegetables that exhibit common characteristics; for example, they are farmed on a global scale and exhibit a wide range of applications, including fresh consumption and use in various food and beverage products. As is frequent, many species or genera share a common name, and this can lead to some confusion when looking for information about a specific variety. In this review, we describe the findings about the biological activity, like antibacterial, antiviral, antidiabetic, and anticancer properties, of two genera of this family, *Cucumis* and *Momordica*, which have been characterized and evaluated in several research studies and regarding which information is readily accessible. Those activities rely on the various physicochemical qualities and nutritional content of each variety, including factors like β-carotene and polyphenols, among others. The goal of this review is to provide a rapid search for each activity examined in the literature, enabling future research on their potential uses in functional foods and nutraceutical supplements.

## 1. Introduction

Cucurbitaceae family members are rich in protein and dietary fiber, which contribute to their health benefits. Additionally, their seeds include essential nutrients such as minerals, copper, phosphorus, zinc, and more [1]. The cucurbit family has antioxidant effects due to several bioactive components, such as cucurbitacins B and E and ellagitannins, additionally, the members of this group possess a high concentration of carotenoids and are abundant in pectin, this characteristic contributes aids individuals with diabetes in managing their blood sugar levels and reducing their reliance on insulin by consuming high-fiber foods [2].

Several plants from the Cucurbitaceae family are edible, either their flesh, seeds, or both, and/or are used for their medicinal effects. Commonly, the word “melon” is used in conjunction with other words (i.e., Bitter melon, Galia melon) to refer to them; however, this term can cause confusion because it can refer to different genera of the family, even though they are all from the same family. This review contains information on the bioactive properties and molecules of the *Cucumis* and *Momordica* species because they are of economic importance and/or have the potential to become a source of bioactive compounds that can be used in functional foods.

### Classification of Genera Cucumis and Momordica

The Cucurbitaceae family comprising around 115 genera and 960 species, is primarily composed of annual herbaceous vines or perennial lianas, commonly having tendrilled structures [3]. The classification of these plants is centered on their morphological, cytological, and floral traits, resulting in two primary subfamilies: Cucurbitoidea and Zanoniodeae. Most cultivars have their origins within the cucurbitoid subfamily, which encompasses 15 tribes and associated genera. This family is characterized by its vast nature, encompassing the Cucurbitaceae. There exist four distinct tribes, namely, Benincasee, Cucurbiteae, Momordiceae, and Sicyoea; among these tribes, the Cucurbiteae group is associated with the cultivation of economically significant fruits, including pumpkins, luffas, melons, and various others [4].

*Cucumis* L., a genus of the Cucurbitaceae family with great nutritional benefits, is commonly consumed fresh and used for multiple dishes like salads, juices, and smoothies [5]; it is also found in pharmaceutical products of vitamin A and C, and used to create facial creams, serums facial masks, shampoos, and hair conditioners [6]. Around the world, it has a high economic value and can grow in various soils due to its great adaptation to different soils and climates [7]. In 2020, it had an estimated production of 42 million tons worldwide [8]. The current varieties of this crop arose from the human selection of wild melons that had a bitter and fine pulp. Interbreeding these plants led to the different botanical forms of these cucurbits [9]. An investigation of the wild melon gene sequence indicated that it originated in India, and was spread to the Middle East and Europe, to be brought to America in the fourteenth century [10]. The classification of *Cucumis melo* has a historical background, and efforts have been made to streamline it. Notable authors such as Naudin, Munger, Robinson, and Pitrat have generated controversy through their systematic study of the subspecies. The placement of various groups within the two subspecies is a subject of disagreement, which is to be expected due to the subjective nature of classification [9,11,12,13,14,15].

Momordica species are cultivated in tropical regions. Tribes and disadvantaged agricultural groups grow it in specific macrogeographic locations [16]. Momordica, belonging to the cucurbitaceae family, encompasses a total of 47 species and is endowed with a bitter taste due to the presence of alkaloid phytochemicals. The assortment comprises different cultivars, including *M. charantia*, *M. muricata*, and *M. dioica*. In India, medicinal herbs are used to a greater extent due to their advantageous properties [17]. *Momordica dioica* is a vegetable with limited economic worth and minor usage; however, some substances with better nutritional value than several regularly consumed vegetables have been researched, and the *Momordica dioica* plant contains alkaloids, glycosides, steroids, triterpenoids, flavonoids, ursolic acid, vitamins, minerals, and fiber, which may treat asthma, excessive salivation and inflammation caused by insect or snake bites, fever, and mental, dermatological, and digestive disorders [18].

Subsequently, Table 1 provides an overview of the taxonomic details pertaining to the members of the Cucurbitaceae family that are examined in this study.

## 2. Genus *Cucumis*

### 2.1. Cucumis metuliferus

Kiwano or horned melon (*Cucumis metuliferus*) is an annual herbaceous vine that thrives in the tropics; it is commonly known as horned melon and grows in Nigeria, South Africa, and other tropical regions [21]. The seeds and pulp are ingested for nutritional and therapeutic purposes, and the pulp has been documented to be rich in numerous nutrients and phytochemicals [22]. Young *C. metuliferus* fruit is dark green with light green speckles; as it ripens, it turns vivid orange with pointed spines, and its interior contains a multitude of green, translucent, slightly mucilaginous juice-sacs with many tightly packed, flat seeds [23]. The fruits’ flavor is a cross between lemons and bananas, and they can be kept at room temperature in arid locations for several months without rotting [24]. It was found that the fruit and seed of *Cucumis metuliferus* are rich in dietary fiber, vitamin C, and antioxidants, all of which have potential health benefits [25]. The reported antioxidant activity of carotenoids confirms, despite the low value, that the *Cucumis metuliferus* seed extract is an excellent source of therapeutic compounds [26]. The seeds contain an oil with a high proportion of unsaturated fatty acids, which is rich in carotenoids and tocopherols, and the peel has pharmacological properties, as it contains rutin, miricetin, and quercetin, which are polyphenolic compounds belonging to the chemical category of flavonoids [27].

#### 2.1.1. Immunological Activity

As a pectin polysaccharide, an investigation revealed that *Cucumis metuliferus* peel polysaccharide (CMPP) might help with immunity enhancement by promoting cell proliferation and increasing the production of cytokines (Nitric Oxid, TNF-α, and IL-6) on RAW264.7 macrophages, indicating that CMPP possesses potential immunological activity and prebiotic effects [28]. The total polyphenol content in the seeds of *C. metuliferus* was higher than in other plant parts, and the peels demonstrated a strong ferrous ion-chelating capacity [29]. The pulp possesses an elevated level of mineral salts, including potassium salts, and is low in sodium salts; at the same time, rutin and lutein with antioxidative, anti-inflammatory, and blood platelet aggregation-inhibiting properties were discovered [30]. A study conducted on mice administered a dosage of 1000 mg/kg of *Cucumis metuliferus* revelead a notable increase in red blood cell, platelet, hemoglobin, and packed cell volume measurements compared to the control group, however, there was a reduction in white blood cell (WBC) count that was dependent on the dosage [31]. The antiviral activity of the alkaloidal components of the fruit extract of *Cucumis metuliferus* E. Meye was examined in chicks infected with Newcastle disease; the results indicated that 600 mg/kg of the extract suppressed the clinical symptoms of viral infection in the chicks [32].

Another study revealed that in chicken embryo fibroblast cells and embryonated chicken eggs induced with infectious bursal disease virus (IBDV), flavonoids extracted from the fruit pulp of *Cucumis metuliferus* had antiviral properties at concentrations of 100 to 0.195 mg/mL, indicating that flavonoids are safe for chickens and have antiviral activity against IBDV [33]. The extracts extended the mean survival time of *P. berghei*-infected mice in contrast to the untreated control group; the highest peak chemo suppression was observed in chloroform extracts of *C. metuliferus* with 98.53%, and this is a potential source of antimalarial templates [34].

#### 2.1.2. Hypoglycemic Activity

It was determined that the fuit extract of *Cucumis metuliferus* may have a hypoglycemic effect on rats with alloxan-induced hyperglycemia but has no effect on the blood glucose curves (BGCs) levels of normoglycemic rats based on an analysis of the BGCs of rats with normoglycemic and alloxan-induced hyperglycemia [35]. *C. metuliferus* extract could lower postprandial glucose levels by inhibiting the activities of β-glucosidase or α-amylase, which are key enzymes in the digestion of complex carbohydrates into adsorbable monosaccharides [15]. The hydro-ethanolic extract of *Cucumis metuliferus* contains ursolic acid, which exhibited similar antioxidant, anti-inflammatory, and antidiabetic effects, in addition to the hydroethanolic extract. *C. metuliferus* demonstrated substantial enzymatic inhibitory activity on diabetes-related enzymes (α-amylase and β-glucosidase) [36].

#### 2.1.3. Anti-Inflammatory and Other Activities

Due to its high phenol and flavonoid content, the seed’s ability to avoid heat-induced albumin denaturation and erythrocyte hemolysis, in addition to protection against proteinase activity, exhibits anti-inflammatory properties [27]. The methanolic extract of the CM fruit could alleviate or decrease nephrotoxicity in cockerels at doses between 400 and 600 mg/kg [37]. The impact of methanol extract on total WBC in cockerels was examined and all treated groups reported a substantial rise in the value of total WBC by day 14, while those receiving 600 mg/kg had a significant increase by day 7 [38].

### 2.2. Cucumis agrestis

The fruit measures 25 by 20 cm and is small, round, or oval, light green to brilliant yellow, with a crunchy exterior and a mildly sweet and sour taste [39]. This is typically consumed as a vegetable, as it is known as small gourd, wild melon, wild musk, chibber, and kachri. Originating in Africa, tropical America, and Southeast Asia, this plant possesses a multitude of phytoconstituents that contribute to its therapeutic potential, these constituents consist of alkaloids, tannins, flavonoids, carbohydrates, proteins, glycosides, steroids, triterpenoids, and phenolic acids [40]. According to the literature, *Cucumis melo*. var. *agrestis* is frequently consumed as a vegetable un rural communities, which explains why its commonly referred to as “wild musk melon” because of its vine-like, untamed development [39].

A study proposes using many seeds to produce biodiesel as an alternative use [41]. An investigation employing the hydroalcoholic leaf extract of *Cucumis melo* var. *agrestis* quantified the total phenolic content and total flavonoid content using a UV-spectrophotometer, and the results indicated that the total phenolic content was 77.82 mg/g of extract calculated as Gallic acid equivalent and the total flavonoid content was 30.06 mg/g of extract calculated as Gallic acid equivalent [42]. The seeds of this fruit are widely used. In a study using the Folin–Ciocalteu method, the total phenolic content of oil seed was determined to be between 33.0 and 31.9 mg/g of dried product, and the phenolic compounds identified using high performance liquid chromatography with a diode array detection were the catechin vanillic acid, sinapic acid, and calistephin [43].

#### 2.2.1. Antihyperglycemic Activity

The hydroalcoholic extract of *Cucumis melo agrestis* leaves (HALEC) was examined for its antihyperglycemic and antihyperlipidemic effects in streptozotocin (STZ)-nicotinamide (NIC)-induced diabetic rats, and it was shown that in glucose-loaded normal rats, the concentration of blood glucose significantly diminished 120 min after HALEC administration, indicating that HALEC has an effective antidiabetic activity [44]. Using an alloxan-induced diabetic rat model, the ethanolic extract was tested for its antidiabetic potential and found to be active in comparison to the standard drug pioglitazone. Diabetic rodents that were treated demonstrated a substantial reduction in plasma insulin levels when compared to the normal control group, this finding suggests that the treatment with extract effectively restored plasma insulin levels to approximately normal levels [45].

#### 2.2.2. Antioxidant Effect

Researchers studied the chemo-preventive effect of the methanolic fruit extract of *Cucumis melo* var. *agrestis* (MECM) on diethyl-nitrosamine-produced hepatocellular carcinoma in Sprague Dawley rats, where the results determined that the MECM was clearly lacking the capacity to act as a chemo-preventive agent; however, its moderate antioxidant effect might place it as an effective adjuvant for the treatment of hepatocellular carcinoma [46]. Another investigation evaluated the ability of *Cucumis melo* var. *agrestis* seed extract to scavenge hydrogen peroxide. The pulp extract exhibited significant effects in the range of % scavenging activity from 66 to 112%, which showed that *Cucumis melo* var. *agrestis* fruits effectively demonstrated in vitro antioxidant activity [47]. The antioxidant capacity of methanolic extracts of *Cucumis melo* var. *agrestis* were evaluated in a quantitative analysis, using the 2,2-diphenyl-1-picrylhydrazyl-hydrate (DPPH) method, and the results showed that the highest radical scavenging activity of methanolic extract was 75.59% at a concentration of 300 µg mL^−1^ [43].

### 2.3. Cucumis melo L.

*Cucumis melo* contains a high concentration of polyphenols associated with potential health, cardiovascular, diuretic, digestive, and antiparasitic benefits [5]. It contains carotenoids, in particular β-carotene, which provide the orange color in the pulp as well as provitamin-A activity. These have antioxidant potential, which decreases the risk of comorbidities associated with obesity, such as cardiovascular diseases and cancer [48]. In addition, *Cucumis melo* is recognized for being rich in vitamin C, vitamin E, polyphenols, carotenoids, and phytochemicals [49]. Also, a study revealed the presence of several biologically active phytocompounds in *Cucumis melo* extracts obtained with various solvent systems, demonstrating the fruit’s cancer-inhibiting properties, and supporting the fruit’s use in future research for the treatment of various diseases [50]. The fruit is used for its juice, with thermal sterilization techniques, such as high-temperature and short-time sterilization, commonly employed to increase safety and expiration life [51]. According to study results, the peels possess proteolytic (4.24 U/mg protein) and milk-clotting (6300 One Milk-Clotting Unit/mg protein) activities, with a milk-clotting activity/proteolytic activity ratio of 1485. Further, biological precipitation with carrageenan enhances the biological activity of the retrieved proteins in peels, demonstrating the potential use of peel juice as a vegetable rennet and the synthesis of bioactive peptides [52]. In Africa, oil extracted from *Cucumis* seeds is utilized for food preparation, whereas in India, *Cucumis* is cultivated and consumed as a vegetable. Wines have been produced from the alcoholic fermentation of melon cultivars Jimbee (smooth and yellow skin with orange pulp) and Okashi (yellow-orange-red skin with pale green pulp) [53].

#### 2.3.1. *Cucumis melo* var. *cantalupensis*

**Figure 1:**
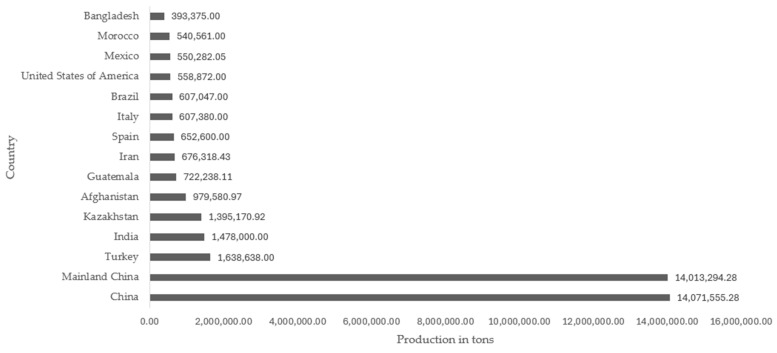
Top 15 cantaloupe melon and other varieties producing countries in the world [55].

A popular fruit known as rock melon is currently seeking a new use for its skin, which has no economic value and is discarded as refuse [54]. The epidermis of the cantaloupe melon is net-like, gray-to-green, or light brown, and faintly ribbed. It is one of the most widely consumed cucurbits in the world due to its richness, juiciness, pleasant flavor, and nutritional value [55]. The *Cucumis melo* L. var. *cantalupensis* cultivar has a world production of 42.6 million tons, and the country with the highest production was China with 14.07 million tons. The commercial value of cantaloupe and other varieties of melon produced worldwide was 12.80 million dollars in 2021, according to data from FAOSTAT [56]. The countries with the highest production of cantaloupe and other varieties of melon worldwide are illustrated in Figure 1.

Furthermore, in terms of its agricultural significance, this plant has a brief generation period and a high degree of phenotypic polymorphism, particularly in its vegetative and fruit morphology [57]. Nutrients like vitamin A, vitamin C, and micronutrients such as potassium and magnesium are abundant in cantaloupe [58]. Cantaloupe is an excellent source of amino acids, particularly neurotransmitters, precursors of nitric oxide, and essential amino acids [54].

In an investigation, the quantified phenolic acids and flavonoids in the extract of the peel were gallic acid (2.45 ± 0.08 mg/g), ellagic acid (0.57 ± 0.01 mg/g), and kaempferol (0.32 ± 0.03 mg/g), and the seed extract contained the highest concentrations of ferulic acid (1.51 ± 0.02 mg/g), kaempferol (0.54 ± 0.02 mg/g), and gallic acid (0.07 ± 0.02 mg/g) [59]. The phenolic content and antioxidant activity of methanolic extracts were also examined: the skin extract showed the highest total phenolic content at 8.47 mg GAE/g extract (expressed as milligram of gallic acid equivalent per gram of extract), and the total flavonoid content in the skin extract was 5.23 μg RE/g extract (represented as rutin equivalent per gram of extract). These findings indicate that methanolic extracts of cantaloupe skin might work as a natural antioxidant for dietary and nutraceutical applications and could be used to inhibit lipid auto-oxidation [60].

#### 2.3.2. Anti-Inflammatory Effect

This fruit variety is abundant in carotenoids that contain vitamin A. Previous studies have shown carotenoids with pro-vitamin A activity (β-carotene, α-carotene, and β-cryptoxanthin), and its derivatives may perform specific functions in mature adipocytes, such as regulating metabolism, the production of inflammatory mediators, and thus oxidative stress; consequently, this fruit is of fundamental significance for the nutritional status of obesity [61]. In a recent study, nanoparticles containing a crude carotenoid extract from cantaloupe melon showed minimal toxicity and a more favorable appearance in the liver and intestines of an experimental model of chronic inflammation [62].

An in vivo study compared the ethanolic extracts of *Cucumis melo* var. *cantalupensis* peels (CCP) and *Cucumis melo* var. *cantalupensis* pulps (CCU) with those of *Cucumis melo* var. *reticulatus* peels (CRP) and *Cucumis melo* var. *reticulatus* pulps (CRU) at concentrations of 25 and 50 mg/kg. After 3 h, all extracts greatly reduced the carrageenan-induced increase in the edema volume of rat paws, but CRU at 50 mg/kg showed a 69.41% inhibitory effect, while CCP caused a 37.90% inhibition of edema development [63].

#### 2.3.3. Anticancer Property

A study revealed that oral supplementation with *Cucumis melo* superoxide dismutase (SOD) and wheat gliadin could indicate a significant improvement in quality of life, as tumor cells exhibit decreased SOD activity, and the overexpression of this enzyme can reduce the incidence of cancer [64]. Cucurbitacin B from *Cucumis melo* var. *Cantalupensis* controls lung cancer cell proliferation and apoptosis by inhibiting the inflammatory process (IL-6/STAT3) pathway via the specific transcript ARN lncRNA XIST/miR-let-7c axis [65].

### 2.4. Cucumis melo var. reticulatus

The Galia melon represents one of the most widely grown crops in Spain, due to its highly valued sensory qualities, and it has the potential to be used as a primary material in the fresh-cut or minimally fresh processing industries [66]. It is widely used worldwide, and studies have shown that its seeds also have antioxidant, antiproliferative, and probiotic properties, as well as significant amounts of phenolic compounds, flavonoids, minerals such as magnesium, phosphorus, sodium, and potassium, polyunsaturated fatty acids, and essential amino acids, such as methionine, isoleucine, tyrosine, phenylalanine, and valine [67]. Its antioxidant effects are described below.

The extracts obtained from the fruit residues (peel and seeds) demonstrated antioxidant activity and adequate metal chelating capacity, which may be construed as an antioxidative property [7]. In a study comparing distinct kinds of Galia melons, nonorganic Galia melon from Honduras/peel contained the most 3-hydroxybenzoic acid, a substance found in grapefruit and olive oil with properties like antifungal, antimutagenic, and antimicrobial [68]. Butyl acetate, 2-methyl-butyl acetate, and hexyl acetate have been identified to be the most abundant compounds in Galia-type melons, and it seems probable that these volatiles could be used to enhance the sensory quality of melons in addition to solids that are soluble [69].

### 2.5. Cucumis melo L. inodorus

There are several varieties within the *Cucumis melo* L. *inodorus* category, like Honey Dew, Canary, Crenshaw, Ivory Gaia, and Honey Dew. The nutritional composition of fresh *Cucumis melo* L. *inodorus* fruit per 100 g edible fraction (rejecting 54–49% rind, 5% cavity components) was: Moisture 89.82 g, energy 150 kJ, (36 kcal), protein 0.54 g, fat 0.14 g, ash 0.41 g, carbohydrate 9.09 g, total dietary fiber 0.8 g, and total sugars 8.12 g [70]. *Cucumis melo inodorus* is an excellent source of vital nutrients and minerals, such as magnesium, potassium, iron, vitamins C, A, and B_6_, calcium, pantothenic acid, omega-3, omega-6, and zinc [71]. This fruit belongs to the group of well-known winter melons. The absence of netting on these melons results in a diminished aroma, albeit accompanied by an exceptionally prolonged shelf life [72]. One example of *inodorus* is Snow Leopard, which is an Ivory Gaya melon with an average fruit weight of 2.5 lb, a white exterior with a honeydew melon-like flavor, and a high yield of 5–6 fruits per plant [73]. Another variety of this melon is the Canary, which is a vibrant yellow fruit with a white interior [74]. Also called sweet melon or golden melon, its interior is pale green in color and has a sweet flavor. Because of its potassium content, it has cardioprotective properties. Likewise, studies with mice conclude that it is a fruit that may have benefits in managing insulin resistance and the inflammation of adipose tissue [75].

**Figure 2:**
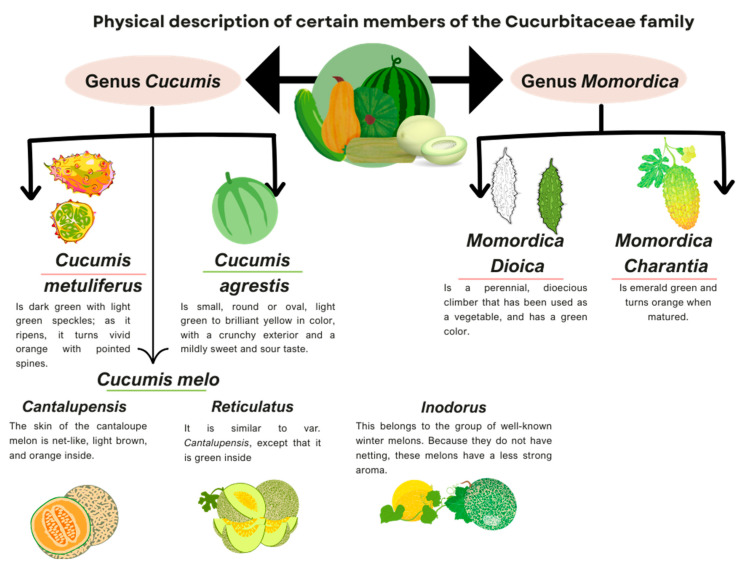
Physical description of the members of the Cucurbitaceous family discussed in this article. Information adapted from: [23,38,54,65,71,77,106].

Most studies on this type of cucurbit focus on the characterization and information about their compounds, nevertheless, the available information regarding the therapeutic efficacy of these compounds in specific diseases is insufficient. In Figure 2, the physical characteristics of the types of the fruits described here are summarized.

A significant portion of the available data about the species within the genus *Cucumis* focuses on the nutritional composition, and the preference for fresh consumption in certain preparations, such as juices or smoothies, that can be attributed to the fresh and flavorful pulp of the fruit. Nevertheless, it is worth noting that some fruits possess compounds that have potential either for extraction or to produce functional foods. It is crucial to keep in consideration that the preservation of these fruits is challenging due to their high water content, approximately 95%. As a result, their consumption is often restricted, leading to significant losses of these fruits, which contain valuable molecules with interesting bioactivities. Therefore, further research is necessary to understand the properties of these fruits and explore methods for their preservation or the creation of functional foods.

## 3. Genus Momordica

### 3.1. Momordica charantia

The bitter melon is a plant that is cultivated in tropical and subtropical areas. It has carbohydrates, proteins, fibers, vitamins like C, A, E, B_1_, B_2_, B_3_, B_9_, and minerals calcium, potassium, magnesium, zinc, iron, and phosphorus [76]. The chemical composition of bitter melon is rich in phenolic compounds such as gallic acid, tannic acid, catechin, caffeic acid, p-coumaric acid, ferulic acid, and benzoic acid [77].

All portions of the plant, including the fruit, are extremely acrid, living up to its common names “bitter melon” or “bitter gourd”. The fruit of *M. charantia* (MC) is oblong or spindly with bumps and resembles a small cucumber. The young fruit is emerald green and turns orange when matured, while the flesh transforms from white to crimson as it matures. In various areas of the world, the fruit is consumed as a vegetable at all stages between maturation and ripeness [78]. This plant, which is used in herbal medicine, shows multiple pharmacological properties, including antidiabetic, anthelmintic, antimalarial, and laxative properties; it also treats eczema, dysmenorrhea, gout, jaundice, leprosy, hemorrhoids, pneumonia, psoriasis, rheumatism, and scabies [79].

#### 3.1.1. Anti-Inflammatory and Antioxidant Activity

Previous studies have shown that restraint stress can increase serum transaminase activity and hepatic nitric oxide (NO) content [80]. Numerous studies have examined the hypoglycemic and anti-obesity properties of *M. charantia*. Both the mechanism of action and its material foundation are unknown [81]. However, one study examined the hepatoprotective effect of *Momordica charantia* water extract (MWE) against liver injury in rodents subjected to restraint stress [82].

A study suggested that neuronal cells are protected from oxidative stress-induced cell damage by polyphenolic compounds, which are extracted from natural products; therefore, it is relevant that *M. charantia* studies reported therapeutic efficacy, such as decreasing hepatic gluconeogenesis and increasing hepatic and muscle glycogen content [83].

Chronic systemic inflammation in diabetic patients contributes to an increase in blood glucose levels and is a risk factor for cardiovascular disease and obesity. Chronic inflammation is associated with the pathogenesis of numerous diseases, including neurodegenerative disorders, cardiovascular disease, obesity, metabolic syndrome, type 2 diabetes, and cancer [84]. In a study, *M. charantia* extracts decreased the expression of intercellular adhesion molecule and tumor suppressor (miR-221/222) in tumor necrosis-factor-alpha-treated mouse lung tissues, while reducing factors with a critical role in biological processes, such as immunity, inflammation, cell growth and survival, and development (PI3K/Akt/NF B/IB). Therefore, the administration of MC extracts prior to tumor necrosis factor alpha (TNF-α) suggests that supplementation with bitter melon might be advantageous as a chemo-preventive agent for individuals at risk of inflammatory-related diseases [85]. Another study showed that glucan endo-1,3-beta-glucosidase (BG-4), a 4 kilodaltons (kDa) peptide extracted from bitter gourd seeds using 70% ethanol, has exceedingly strong trypsin-inhibiting activity [86]. The findings of a murine study (RAW2642) investigating the impact of *M. charantia* on impaired glucose metabolism induced by lipopolysaccharide (LPS) revealed that *M. charantia* reduced the expression of inflammatory genes including Interleukin 6 (IL6), TNF-α, Interleukin 1 (IL1), cyclooxygenase-2(COX2), Inducible nitric oxide synthase (iNOS), and Interleukin 10 (IL10) [87]. Bitter melon extract (BME) inhibited IL-1 mRNA expression in the head kidney, spleen, and intestine of common carps, indicating that BME is a positive expression in inflammatory cytokines [88].

#### 3.1.2. Anti-Obesity and Antidiabetic Activity

*M. charantia* contains phenolic compounds that have been reported to exhibit potential beneficial effects on obesity in animals. It was observed that rodents administered a dose of a dose of polyphenol (100 mg/kg Body Weight) had significantly reduced body weight and serum total cholesterol levels compared to the control group (without treatment) thirty days later [89]. The ethanolic extract of *M. charantia* improved the functioning of B cells and insulin levels in neonatal streptozotocin (STZ)-induced type 2 diabetic rodents [90]. Among the antidiabetic effects of rutin in MC is the inhibition of carbohydrate absorption from the small intestine, the stimulation of insulin secretion from cells, and the protection of the islets of Langerhans from degenerative processes [91].

A study isolated a novel insulin receptor-binding protein from *Momordica charantia*, identified a specific sequence of 19 amino acids (mcIRBP-9), and confirmed the peptide’s gastric resistance and hypoglycemic activity [92]. *M. charantia* has been shown to increase insulin sensitivity via multiple mechanisms observed in animal models, including an increase in the rate of phosphorylation of the insulin receptor substrate [93]. Yi-Sun Yang showed that the consumption of retinol-binding interphotoreceptor protein (mcIRBP-19-BGE) capsules at 600 mg/day for three months might reduce Glycosylated Hemoglobin (HbA1c) by about 0.5% in a subset of individuals whose hypoglycemic medications did not have any effect on HbA1c reduction [94].

#### 3.1.3. Antibacterial Capacity

An investigation suggests that *M. charantia* leaves could serve as a viable alternative antibacterial agent against *K. pneumoniae* [95]. Due to its outstanding antibacterial properties against both Gram-positive and Gram-negative bacteria, MC extract is also a suitable and sustainable replacement for antibacterial drugs, and the WST-1 assay, which examined cell proliferation and cell viability proved that MC extract is suitable for applications such as wound dressings [96]. The maximum zone of inhibition of 28.3 ± 1.2 mm was observed for the ethanolic extract of *M. charantia* against poultry-associated bacteria such as *B. licheniformis* [97].

*M. charantia* extracts had an antibacterial effect towards pathogenic bacteria isolated from ready-to-eat foods; however, water extracts proved that they were stronger in comparison with ethanol extracts, exerting significant inhibitory activities against most of the isolates [98]. Variations of *M. charantia* var. *charantia* and var. *muricata* (VC and VM) are a rich source of phenolic compounds. According to the results of an antibacterial assay, var. *muricata* exhibited antibacterial activity against *E. coli*, *K. pneumonia*, *P. aeruginosa*, *M. luteus*, and *S. aureus*, whereas var. *charantia* revealed antibacterial activity against only three species of microbes like *S. aureus*, *P. aeruginosa*, and *E. coli* [99]. The leaf extract of bitter melon (*Momordica charantia* L.) has antibacterial activity which is antagonistic toward *A. hydrophila* with an intermediate level of resistance [75].

#### 3.1.4. Anticancer Property

*M. charantia* extract or compounds exhibit anticancer activity by interacting with and penetrating the cell membrane of breast cancer cells, according to one study; however, the precise mechanism by which this occurs remains unknown [100]. *Momordica charantia* L. oligopeptides (MCLO-12) showed diminished cytotoxicity towards normal human lung fibroblast cells [101]. In the tumor tissues of bitter melon juice (BMJ)- and bitter melon extract (BME)--fed mice, the percentage of antigen CD-31-positive cells significantly decreased [102]. The quantity of colon cancer cells (WiDr) may decrease while the proliferative activity lowers by MC extract, according to a proliferation test which revealed that the extract inhibited the proliferation rate in a 24-hour incubation period [103]. Another investigation concluded that during 48 h of treatment in breast cancer cell lines in vitro, the MC ethanol extract and Kuguacin-J (K-J), which is an effective chemosensitizer for treating tumors resistant to multiple drugs, appeared to marginally increase cell viability when compared with K-J and cisplatin, which killed the cell line for normal human breast cells (MCF-10A), with cisplatin being more damaging at both low and high doses than K-J at the higher concentration [104].

A study revealed that MC extract acts as a potentially activated protein kinase (AMPK) activator by increasing AMPK via Ca^2+^/calmodulin-dependent protein kinase-β, suppressing mechanisms and signs associated with cell survival and development to promote apoptosis and preventing progression and metastasis of ovarian cancer, as well as being used as an adjunct to enhance the efficacy of cisplatin-based chemotherapy in ovarian carcinoma [105]. The results of another study indicate that the ethanol fruit extract of *M. charantia* has the most prevalent cytotoxic activity of all the extracts examined (>80%) against Jurkat cell lines; these results suggest that the extract could serve in the development of anticancer drugs against lung cancer, breast cancer, chronic myeloid leukemia, and T cell leukemia [106].

### 3.2. Momordica dioica

*Momordica dioica* is a perennial, dioecious climber that has been used as a vegetable for thousands of years despite having a higher nutritional value than many commonly ingested vegetables [107]. It has various names, including spine gourd, akakara, bodakakara, kakor, teasle gourd, kantola, and kakrol, and its distribution is primarily in India, Sri Lanka, Myanmar, and Bangladesh [108]. The total phenolic content of *Momordica dioica* Roxb. is approximately 9.25 mg/GAE per gram of dried sample, and it has 2.68% fat, 12.29% crude fiber, and 67.14% carbohydrates [109].

*M. dioica* possesses a variety of phytoconstituents, bioactive compounds with multiple benefits that are essential in medicinal remedies, likewise is used as an insecticide, in addition to being a protectant against *Callosobruchus chinensis* in crops [110].

#### 3.2.1. Anticancer Property

Cucurbitacin triterpenoids of *Momordica dioica* Roxb. fruit has demonstrated dose-dependent antitumor activity against Ehrlich Ascites carcinoma (EAC)-induced liquid tumors, analogous to that of cisplatin [111]. The MTT assay, which indicates cell viability, revealed that a *Momordica dioica* peptide inhibited the viability of cells in a dose-dependent manner [112]. And the results of a study indicate that the aqueous fruit extract of *Momordica dioica* exhibited anticancer properties by inhibiting ovarian carcinoma (PA-I) and human cervical cancer (Hela cells) by 50% at an IC50 concentration of 40 g/mL [113]. The purification and isolation of proteins from *Momordica dioica* have paved the way for the advancement of peptide-based drug delivery. It is apparent that the anti-proliferative proteins isolated from the seeds of *Momordica dioica* can be treated with enzyme digestion, which results in different peptides with antiproliferative activity of varying molecular masses [114].

#### 3.2.2. Other Effects on Health

The hydroalcoholic extract of fruits of *Momordica dioica* (HAEMD) and the aqueous extract of leaves of Lagerstroemia speciosa were evaluated for potential antifertility effects in experimental rats, with the conclusion that HAEMD possessed anovulatory and estrogenic properties [115]. In a study comparing the antibacterial and antioxidant activity of the root and fruit extracts of *Momordica charantia* L. and *Momordica dioica* Roxb, the antibacterial capacity of the extracts was confirmed, with the antibacterial activity of the fruit extract being greater than that of the root extract for both Gram-negative and Gram-positive bacteria. Additionally, *M. charantia* and *M. dioica* inhibited 2,2-diphenyl-1-picrylhydrazyl (DPPH) free radicals, demonstrating potent antioxidant activity [116]. Treatment with the ethanolic extract of *M. dioica* in rats with type 2 diabetes modified glycemic and lipidemic conditions, and decreased fasting serum glucose, cholesterol, and triglyceride levels; however, there are no panoptic reports on the primary active phytoconstituents of *M. dioica* ethanolic extract, so establishing which are responsible for the antidiabetic activity is difficult [117]. Another study provides information concerning the anticipated effectiveness of flavonoids and triterpenoids from the *M. dioica* plant against coronavirus proteases and proteases with comorbidities [118].

The species of the genus Momordica have received more extensive research on their bioactivities and molecular composition compared to the fruits of the genus Cucumis. This is mostly due to their greater utilization of herbal medicine. Typically, bitter, or acidic flavors are indicative of beneficial health attributes, as they are mostly derived from molecules possessing biological activity. While certain fruits, like *M. dioica*, are consumed as part of a diet, their food distribution is not as widespread as that of the Cucumis genus. However, they have the potential to serve as a source of biomolecules with health-promoting properties. These biomolecules can be obtained through various methods, such as fresh consumption, oil extraction, or the extraction of molecules for other purposes.

Figure 2 below illustrates the physical characteristics of the Cucurbits discussed in this article.

Finally, a summary of this review is shown in Table 2.

## 4. Conclusions

The Cucurbitaceae family has a wide range of traits that contribute to their health advantages. These properties can be attributed to several components, including significant amino acids, vitamins, fibers, and secondary metabolites such as triterpenoids, alkaloids, polyphenols, and other proven substances. Nevertheless, further investigation is required to examine the potential of each molecule in various illness contexts, including cardioprotective and hepatoprotective properties, among others. Due to the extensive and diverse members of the Cucurbitaceae family, there is a significant research gap in this area. Some species are still undergoing characterization studies and plant studies, and there is currently no research on the health applications of their components. Nevertheless, some species are known for their properties in communities and are used in herbal medicine. However, further studies are required to scientifically confirm their effects for health applications. While several species have been extensively investigated, there are still additional variations that require more exploration. Furthermore, it is worth noting that a greater number of antiviral investigations have been conducted on *Momordica* species in earlier years, whereas the studies on *Cucumis* species have been conducted more recently. The genus *Cucumis*, despite being widely consumed for its appealing sensory qualities, does not exhibit the same level of bioactivities as the plants belonging to the genus *Momordica*. Due to its familial similarity, diverse range, and global distribution, there exists potential for variation in the nutritional composition of fruits. While certain compounds have already been identified, it is acknowledged that variations may arise from the plant’s growth state and environmental conditions. Consequently, further investigation, both in vitro and in vivo, is necessary to explore novel food products or nutraceuticals derived from less commonly consumed fruits, such as *Momordica* species. Exploration exhibits the potential for enhancing health in individuals with distinct diseases. It is crucial to acknowledge the potential for extracting bioactive chemicals from crops that are already being consumed, particularly those belonging to the *Cucumis* genus, where a significant proportion of fruit is wasted. Conversely, these crops often need specific procedures to maintain their properties, either for consumption in minimally processed foods or as an ingredient in new foods. These procedures aim to preserve or enhance the functional properties of these crops. Finally, both genera have significant potential for the development of functional foods or the extraction of bioactive compounds, and, due to their remarkable adaptability, they can be cultivated in several regions or from a more sustainable perspective. Each cucurbit in its respective region can be utilized to harness its biological properties to improve health.

## Figures and Tables

**Table 1:** Taxonomy of the Cucurbitaceae family members [19,20].

	*Cucumis* *metuliferus*	*Cucumis* *agrestis*	*Cucumis melo*	*Cucumis melo* var. *reticulatus*	*Cucumis melo* var. *inodorous*	*Momordica* *dioca*	*Momordica charantia*
Family	Cucurbitaceae	Cucurbitaceae	Cucurbitaceae	Cucurbitaceae	Cucurbitaceae	Cucurbitaceae	Cucurbitaceae
Subfamily	Cucurbitoideaea	Cucurbitoideaea	Cucurbitoideaea	Cucurbitoideaea	Cucurbitoideaea	Cucurbitaceae	Cucurbitaceae
Tribu	Melothrieae	Benincaseae	Benincaseae	Benincaseae	Benincaseae	Momordiceae	Momordiceae
Genus	*Cucumis*	*Cucumis*	*Cucumis* L.	*Cucumis* L.	*Cucumis* L.	*Momordica* L.	*Momordica* L.
Species	*Cucumis* *metuliferus*	*Cucumis* *agrestis*	*melo.* L.	*melo.* L.	*melo.* L.	*Momordica* *dioca*	*Momordica charantia*
Variety	*Metuliferus*	*Agrestis*	*Cantalupensis*	*Reticulatus*	*Inodorus*	*Dioca*	*Charantia*

**Table 2:** Comparison of bioactivities of Cucurbitaceae family species.

Name	Bioactive Compounds	Functions	Reference
*Cucumis metuliferus*	Dietary fiber, vitamin C, antioxidants, carotenoids, unsaturated fatty acids, tocopherols, rutin, miricetin, lutein, quercetin, and mineral salts.	Immunological, antioxidant, antiviral, anti-inflammatory, hypoglycemic activity, and nephrotoxicity.	[15,25,26,27,28,29,30,31,32,33,34,35,36,37]
*Cucumis agrestis*	Alkaloids, tannins, flavonoids, carbohydrates, proteins, glycosides, steroids, triterpenoids, and phenolic acids.	Antihyperglycemic activity and antioxidant effect	[38,39,41,42]
*Cucumis melo* var. *cantalupensis*	Vitamin A, C, and micronutrients such as potassium and magnesium, amino acids, carotenoids, phenolic acids, and flavonoids.	Anti-inflammatory effect, and anticancer properties.	[57,58,59,60,61,62,63,64]
*Cucumis melo* var. *reticulatus*	Phenolic compounds, flavonoids, and minerals such as magnesium, phosphorus, sodium, and potassium.	Antioxidant, antifungal, antimutagenic, and antimicrobial activity.	[7,66,67]
*Cucumis melo* L. *inodorus*	Phenolic compounds, potassium, iron, vitamins C, A, and B6, calcium, pantothenic acid, omega-3, omega-6, and zinc.	No specific disease research.	[69,70,72,74,118]
*Momordica* *charantia*	Polysaccharides, peptides, proteins, lipids, terpenoids, saponins, and sterols.	Hepatoprotective effect, anti-inflammatory, antidiabetic, anti-obesity, antioxidant, antibacterial, and anticancer activity.	[75,76,81,82,83,84,85,86,87,88,89,90,91,92,93,94,95,96,97,98,99,100,101,102,103,104,105]
*Momordica dioica*	Phenolic compounds, fiber, and carbohydrates.	Anticancer, contraceptive, antibacterial, antioxidant, hypoglycemic, anti-lipidemic, and antiviral activity.	[72,108,109,110,111,112,113,114,115,116,117]
